# Effects of Propofol, a Sedative-Hypnotic Drug, on the Lipid Profile, Antioxidant Indices, and Cardiovascular Marker Enzymes in Wistar Rats

**DOI:** 10.1155/2013/230261

**Published:** 2013-06-06

**Authors:** Oluwatosin A. Adaramoye, Olugbenga Akinwonmi, Olubukola Akanni

**Affiliations:** ^1^Department of Biochemistry, University of Ibadan, 1 University Road, Oyo-Ojoo Way, Ibadan 20005, Nigeria; ^2^Department of Anaesthesia, University College Hospital, Ibadan, Nigeria

## Abstract

In recent years, the activity of anaesthetic propofol on biological processes has been attracting attention. The effect of propofol on biochemical indices in animals is unknown. In this study, we examined the effects of propofol on lipid profile, antioxidant indices, and cardiovascular marker (CVM) enzymes in rats. The study consists of three groups of seven rats each. Group one received corn oil (Control) while groups two and three received propofol (doses of 2 and 4 mg/kg body weight, resp.). Results showed that administration of propofol caused a significant (*P* < 0.05) and dose-dependent increase in the levels of total bilirubin. Propofol at 2 and 4 mg/kg increased the levels of serum total cholesterol by 74% and 55%, triglycerides by 97% and 115%, and LDL-C (low-density lipoprotein-cholesterol) by 45% and 73%, respectively, while HDL-C (high-density lipoprotein-cholesterol) decreased by 41% and 54%, respectively. Propofol significantly (*P* < 0.05) increased the levels of the hepatic reduced glutathione (GSH) and activities of GSH-dependent enzymes. Propofol at 2 and 4 mg/kg increased the activities of CVM enzymes: lactate dehydrogenase by 1.7 and 1.8 folds and creatinine phosphokinase by 2.0 and 2.1 folds, respectively. Taken together, propofol increased the levels of GSH and GSH-dependent enzymes but adversely affected the lipid profile of the rats.

## 1. Introduction

Oxidative stress can be described as an imbalance between the production of free radicals and antioxidant defence. Over the last decades, it has become amply evident that oxidative stress, usually in the form of reactive oxygen species (ROS), is a critical pathogenic factor in the development of several systemic diseases [1, 2]. Therefore, inhibition of ROS formation, scavenging of ROS, or interfering with ROS pathogenic signaling pathways might be the potential ways to protect against oxidative stress-induced ailments [3].

**Figure 1:**
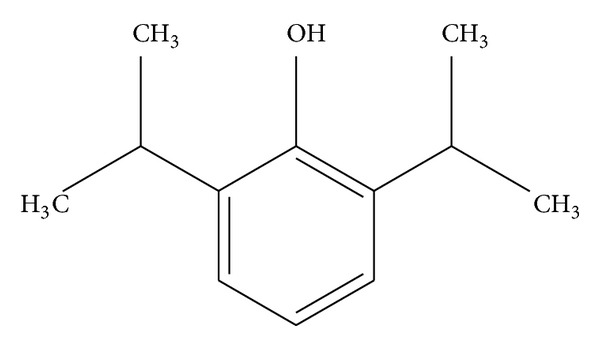
Structure of propofol (2,6-diisopropylphenol).

Propofol (2,6-diisopropylphenol) (Figure 1), a highly lipid-soluble anaesthetic, is widely used for induction and maintenance of general anaesthesia. Propofol ameliorates oxidative injury in several organs, including the heart [4], lungs [5], brain [6], liver [7], and testis [8]. Propofol is chemically similar to the endogenous antioxidant *α*-tocopherol (vitamin E) and, theoretically, it should have similar properties [9]. It is not surprising therefore that many studies have demonstrated antioxidant effects of propofol *in vitro* [10–12] and *in vivo* [13]. Propofol has also been shown to elicit antiapoptotic effect in human umbilical vein endothelial cells by acting as an antioxidant [14]. To the best of our knowledge, the effects of propofol at high doses on the lipid profile, cardiovascular marker enzymes, and enzymatic and nonenzymatic antioxidant indices in rodents have not been determined. This study was therefore designed to evaluate the aforementioned parameters.

## 2. Materials and Methods

### 2.1. Chemicals

Propofol also called Disoprivan by AstraZeneca (Switzerland) used for this study was obtained from one of the coauthors who is an Anaesthetist (Dr. Olugbenga Akinwonmi of the Department of Anaesthesia, University College Hospital, Ibadan, Nigeria). Glutathione, hydrogen peroxide, 5, 5′-dithios-bis-2-nitrobenzoic acid (DNTB), and epinephrine were purchased from Sigma Chemical Co., Saint Louis, MO, USA. Other chemicals were of analytical grade and purest quality available. 

### 2.2. Animals' Protocol

Inbred male Wistar rats weighing between 200 and 220 g were purchased from the Central Animal House located in the Department of Physiology, University of Ibadan, Nigeria. The animals were kept in well-ventilated cages at room temperature (28–30°C), and maintained on laboratory chow (Ladokun Feeds, Ibadan, Nigeria) and water *ad libitum*. Rats handling and treatments protocol conforms to the guidelines of the Faculty of Basic Medical Sciences, University of Ibadan Animals' Ethical Committee as well as the National Institute of Health (NIH publication 85-23, 1985) for laboratory animals' care and use.

### 2.3. Experimental Design

Twenty-one male albino rats (Wistar strain) were randomly distributed into three groups of seven animals each. Animals were given a period of two weeks for acclimatization before the experiment. The first group served as the control and was given corn oil (Vehicle for the drug). The second group received propofol at a dose of 2 mg/kg body weight (equivalent of human therapeutic dose), and the third group received propofol at a dose of 4 mg/kg body weight (twice therapeutic dose). Propofol was prepared with corn oil and was administered intraperitoneally three times in a week for four consecutive weeks. 

### 2.4. Preparation of Serum

Twenty-four hours after the last dose of the drug, the animals in each group were sacrificed by cervical dislocation, and blood was collected from the inferior vena cava of the heart into plain centrifuge tube. Blood was allowed to stand for 1 h and was then centrifuged at 3000 ×g for 15 min in a bench centrifuge to obtain serum. The serum samples were used for the analysis of biochemical indices and lipid profile of the animals. 

### 2.5. Preparation of Tissues

Liver and kidney from the animals were quickly removed and washed in ice-cold 1.15% KCl solution, dried, and weighed. The tissues were homogenized in 4 volumes of 50 mM phosphate buffer, pH 7.4, and centrifuged at 10,000 ×g for 15 min to obtain postmitochondrial fraction (PMF) of the liver and kidney, which were used for assay of the antioxidant parameters. 

### 2.6. Biochemical Assays

Protein levels in the samples were assayed by the method of Lowry et al. [15] using bovine serum albumin as standard. The activities of serum alanine and aspartate aminotransferases (ALT and AST) were assayed by the combined methods of Mohun and Cook [16] and, Reitman and Frankel [17]. PMF lipid peroxidation levels were assayed by the reaction between 2-thiobarbituric acid and malondialdehyde, an end product of lipid peroxides, as described by Walls et al. [18]. PMF-reduced glutathione (GSH) level was assayed by measuring the rate of formation of chromophoric product in a reaction between 5,5^1^-dinitrobis-2-nitrobenzoic acid and free sulphydryl groups at 412 nm as described by Moron et al. [19]. PMF superoxide dismutase (SOD) activity was measured by the nitro blue tetrazolium reduction method of McCord and Fridovich [20]. PMF catalase (CAT) activity was assayed spectrophotometrically by measuring the rate of decomposition of hydrogen peroxide at 240 nm as described by Aebi [21] while glutathione-S-transferase (GST) activity was determined by the method of Habig et al. [22]. PMF glutathione peroxidase was determined according to the method of Rotruck et al. [23]. The activities of serum creatinine phosphokinase (CK-MB) were estimated using immune-inhibition kinetic assay according to the method of Okinaka et al. [24]. Lactate dehydrogenase (LDH) activities in the serum were determined according to the method of Zimmerman and Weinstein [25]. The total bilirubin levels were assayed by the method of Rutkowski and DEBaare [26] while serum creatinine and urea were estimated by the methods of  Jaffe [27] and Talke and Schubert [28], respectively. Serumtriglyceride (TG) and total cholesterol (TC) levels were assayed using commercial diagnostic kits (Randox). For the determination of HDL level, VLDL and LDL lipoproteins were precipitated by addition of phosphotungstic acid and magnesium chloride. After centrifugation, the supernatant containing the HDL fraction was assayed for cholesterol using Randox diagnostic kit. LDL-cholesterol (LDL-C) was calculated using the formula of Friedewald et al. [29]. 

### 2.7. Statistical Analysis

All values were expressed as the mean ± S.D. of seven animals per group. Data were analyzed using one-way ANOVA followed by the post-hoc Duncan's multiple-range test for analysis of biochemical data using SPSS version 10.0 (SPSS Inc., Chicago, IL, USA). Values were considered statistically significant at *P* < 0.05.

## 3. Results and Discussion

Reactive oxygen species (ROS) attack cellular components containing polyunsaturated fatty acid residues of phospholipids, which are sensitive to oxidation [30]. The peroxyl radicals formed by the attack can be rearranged via a cyclization reaction to endoperoxides which are precursors of MDA, which subsequently lead to lipid peroxidation [31]. Lipid peroxidation (LPO) has been implicated in the pathogenesis of several injuries by many toxicants and may be responsible for cell membrane alterations which can result into enzymes leakage in animals [32]. In this study, administration of propofol did not significantly (*P* > 0.05) affect the levels of serum and renal and hepatic MDA in the rats (Table 3), indicating that the metabolism of propofol may not be linked to the generation of free radicals within the animal tissues. This observation is not strange since propofol has a very active phenolic ring within its structure which resembles *α*-tocopherol and may release electron to terminate free radical reactions during its metabolism in the rats [9]. However, Table 2 shows that the levels of serum total cholesterol, triglycerides, and LDL-cholesterol were significantly (*P* < 0.05) increased following administration of propofol. Precisely, propofol at 2 and 4 mg/kg increased the levels of serum total cholesterol by 74% and 55%, triglycerides by 97% and 115%, and LDL-cholesterol by 45% and 73%, respectively. In addition, propofol at both doses significantly (*P* < 0.05) decreased the levels of serum HDL-cholesterol in the rats by 41% and 54%. Lipids are a heterogeneous group containing active metabolic substances that play an important role in the pathogenesis of drugs-induced liver disease. The most common lipid abnormalities during drug toxicity are hypercholesterolemia and hypertriglyceridemia [33], which was confirmed in this study. Also, in propofol-treated rats, LDL-C was remarkably increased in the serum, while HDL-C was found to be reduced. The increased cholesterol level during propofol administration may be linked to increase in alpha-hydroxyl methyl glutaryl CoA reductase activity, which is the rate limiting step in cholesterol biosynthesis [34]. Likewise, the increased triglycerides levels may be due to the increased availability of free fatty acid, glycerophosphates, decreased triglycerides lipase activity, or decreased fatty oxidation. In the present study, hyperlipidemia was confirmed in the serum of propofol-treated rats.

**Figure 2:**
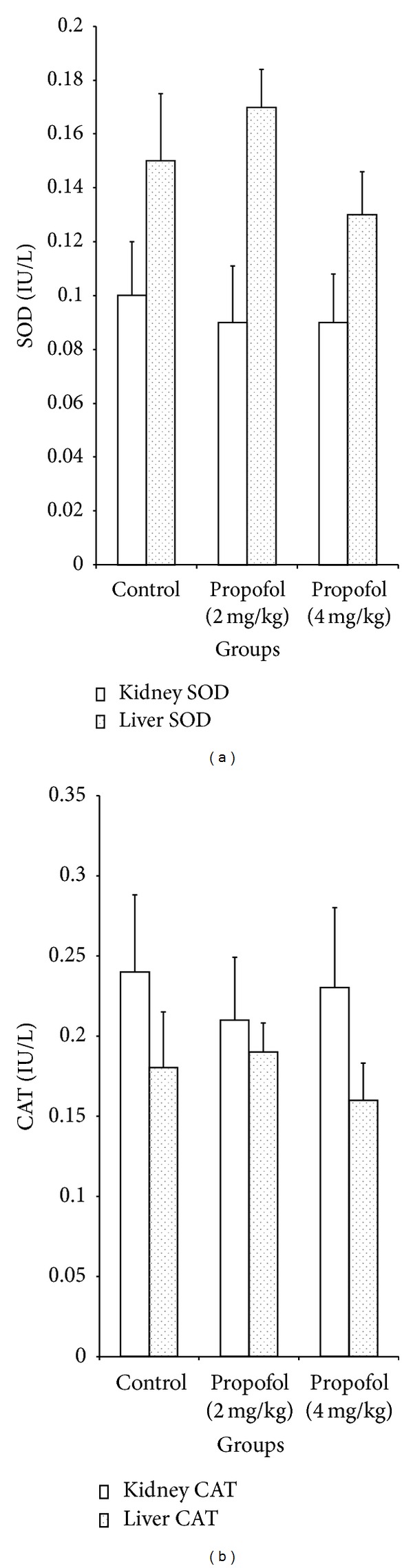
Effects of propofol on the activities of kidney and liver catalase and superoxide dismutase in the rats. SOD: superoxide dismutase, CAT: catalase superoxide dismutase.

**Figure 3:**
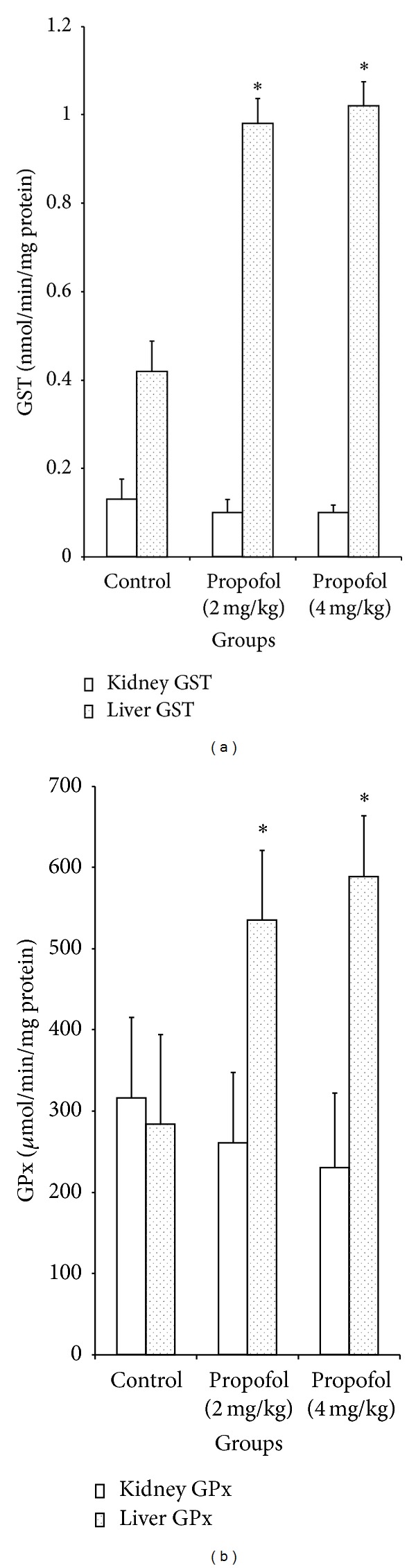
Effects of propofol on the activities of kidney and liver glutathione-S-transferase and glutathione peroxidase in the rats. *Significantly different from control (*P* < 0.05), GST: glutathione-S-transferase, and GPx: glutathione peroxidase.

Propofol at 2 and 4 mg/kg increased the levels of hepatic GSH by 42% and 37%, respectively, when compared to the control (Table 3). In addition, propofol at 2 and 4 mg/kg dose dependently and significantly (*P* < 0.05) increased the activities of hepatic glutathione peroxidase by 1.9 and 2.1 folds and glutathione-S-transferase by 2.3 and 2.4 folds, respectively (Figure 3). However, there were no significant (*P* > 0.05) differences in the activities of renal and hepatic SOD and CAT of propofol-treated rats relative to controls (Figure 2). Reduced glutathione acts as a free radical scavenger and regenerator of alpha-tocopherol, and it plays a significant role in sustaining protein sulfhydryl groups in proteins and other key molecules [35]. In addition, GSH may act as an essential cofactor for antioxidant enzymes, especially GPx and GST [36]. The increase in the levels of hepatic GSH observed in this study may result in increased detoxifying and antioxidant capability of the liver. The GST and GPx are groups of multifunctional proteins that play a central role in the detoxification of electrophilic chemicals and the hepatic removal of potentially harmful hydrophobic compounds from blood [37]. The increased activities of hepatic GST and GPx observed may partly be due to increase in their substrate or induction of *de novo* synthesis of the enzymes. Importantly, propofol increased the levels of GSH and activities of GST and GPx in the liver and thus enhancing the detoxifying potential of the animals.

**Figure 4:**
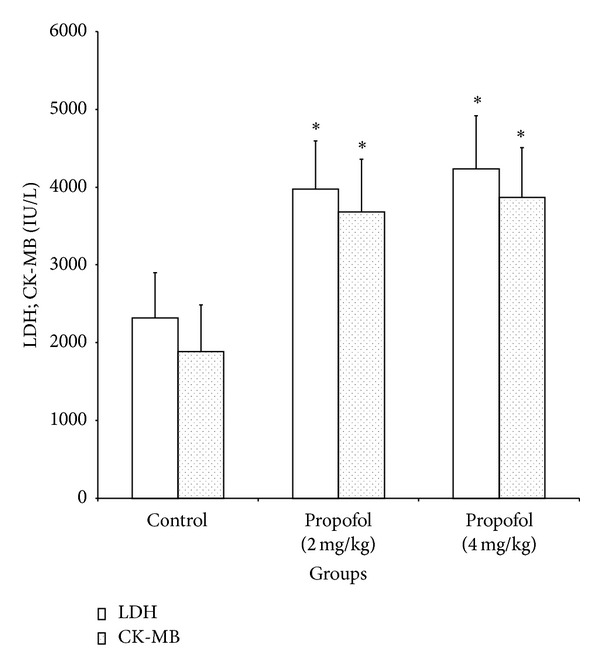
Effects of propofol on the activities of serum lactate dehydrogenase and creatinine phosphokinase of rats. *Significantly different from control (*P* < 0.05), LDH: lactate dehydrogenase, CK-MB: creatinine phosphokinase.

The cardiotoxicity of xenobiotics can be evaluated using the serum activities of marker enzymes especially LDH and CK-MB, which are distributed throughout the body and have isoenzymes that are recognized as markers for muscle and heart lesion [38]. Results from this study showed that propofol at 2 and 4 mg/kg significantly (*P* < 0.05) increased the activities of serum LDH by 1.7 and 1.8 folds and CK-MB by 2.0 and 2.1 folds, respectively (Figure 4). In their studies, Hayden and Tyagi [39] linked the increase in serum CK-MB and LDH of diabetic rats to cardiac muscular damage caused by the disease. Similarly, Wiernsperger [40] stated that the activities of serum CK-MB and LDH are a measure of the state of necrosis in cardiac tissues. In view of the observed increase in CK-MB and LDH activities in propofol-treated rats, the drug may elicit adverse effect on the cardiac tissue.

Administration of propofol at 2 and 4 mg/kg significantly (*P* < 0.05) increased the level of total bilirubin by 52% and 53%, respectively, while serum urea, alanine and aspartate aminotransferases (ALT and AST), and creatinine levels were insignificantly (*P* > 0.05) affected (Table 1). Both AST and ALT are the reliable makers for liver function. It is established that AST can be found in the liver, cardiac muscle, skeletal muscle, kidney, brain, pancreas, lungs, leukocytes, and erythrocytes, whereas ALT is predominantly present in the liver [41]. Also, serum creatinine and urea levels are the sensitive and reliable biochemical indices for evaluation of renal function in animal models [42]. The insignificant effects of propofol on the levels of AST, ALT, and creatinine indicate that the drug has no adverse effect on the hepatic and renal tissues of the rats.

## 4. Conclusions

Our data showed that propofol promotes the antioxidant status of the rats by increasing the levels of reduced glutathione and GSH-dependent enzymes. However, propofol elicited a detrimental effect on the lipid profile resulting in hypercholesterolemia which subsequently leads to abnormally high activities of serum creatinine phosphokinase and lactate dehydrogenase in the rats. The study, therefore, suggests that the use of propofol in patients with cardiovascular or lipid disorders should be carefully reviewed.

## Figures and Tables

**Table 1:** Effects of propofol on serum biochemical indices in the rats.

Grouping	Protein	ALT	AST	UREA	CREAT	TOTAL BIL
	(mg/mL)	(IU/L)	(IU/L)	(*μ*mol/L)	(*μ*mol/L)	(*μ*mol/L)
Control	0.10 ± 0.03	337.9 ± 16.7	565.9 ± 23.4	34.3 ± 5.80	7.57 ± 0.81	56.9 ± 4.80
Propofol (2 mg/kg)	0.11 ± 0.02	346.9 ± 18.4	515.3 ± 33.9	37.6 ± 7.05	7.72 ± 0.66	86.7 ± 6.62*
Propofol (4 mg/kg)	0.12 ± 0.02	359.6 ± 21.3	527.4 ± 44.3	32.8 ± 6.10	7.87 ± 0.64	87.0 ± 8.92*

Values are means ± SD of 7 animals per group.

*Significantly different from control (*P* < 0.05).

ALT: alanine aminotransferase, AST: aspartate aminotransferase, CREAT: creatinine, and TOTAL BIL: total bilirubin.

**Table 2:** Effects of propofol on serum lipid profile of the rats.

Grouping	Serum			
	TC	TG	HDL-C	LDL-C
	(mmol/L)	(mmol/L)	(mmol/L)	(mmol/L)
Control	97.5 ± 3.68	122.1 ± 13.89	28.2 ± 3.55	69.8 ± 5.32
Propofol (2 mg/kg)	169.7 ± 8.42*	240.7 ± 22.36	16.6 ± 2.98*	101.4 ± 5.37*
Propofol (4 mg/kg)	151.3 ± 5.54*	262.0 ± 16.05	12.9 ± 2.68*	124.1 ± 8.03*

Values are means ± SD of 7 animals per group.

*Significantly different from control (*P* < 0.05).

TC: total cholesterol, TG: triglycerides, HDL-C: high-density lipoprotein-cholesterol, and LDL-C: low-density lipoprotein-cholesterol.

**Table 3:** Effects of propofol on nonenzymatic antioxidant parameters in the rats.

Grouping	LPO (nmol MDA/mg protein)			GSH (*μ*g/g tissue)	
	Serum	Kidney	Liver	Kidney	Liver
Control	22.0 ± 3.45	18.5 ± 1.62	18.9 ± 3.55	5.87 ± 1.31	10.1 ± 3.12
Propofol (2 mg/kg)	24.1 ± 4.03	16.2 ± 2.36	19.3 ± 4.16	4.65 ± 0.72	14.3 ± 2.40*
Propofol (4 mg/kg)	21.8 ± 3.01	18.7 ± 1.87	21.0 ± 3.10	5.01 ± 0.63	13.7 ± 2.30*

Values are means ± SD of 7 animals per group.

*Significantly different from control (*P* < 0.05).

LPO: lipid peroxidation, GSH: reduced glutathione.
